# Experimental Study on the Fracture Toughness of Bamboo Scrimber

**DOI:** 10.3390/ma16134880

**Published:** 2023-07-07

**Authors:** Kairan Zhang, Yubo Hou, Yubin Lu, Mingtao Wang

**Affiliations:** 1 School of Advanced Manufacturing, Fuzhou University, Fuzhou 350108, China; 218527124@fzu.edu.cn; 2 Quanzhou Institute of Equipment Manufacturing, Fujian Institute of Research on the Structure of Matter, Chinese Academy of Sciences, Quanzhou 362216, China; houyubo@fjirsm.ac.cn (Y.H.); 2210308@tongji.edu.cn (M.W.)

**Keywords:** bamboo scrimber, fracture toughness, J-integral, numerical simulation

## Abstract

In the past decade, bamboo scrimber has developed rapidly in the field of building materials due to its excellent mechanical properties, such as high toughness and high tensile strength. However, when the applied stress exceeds the ultimate strength limit of bamboo scrimber, cracks occur, which affects the performance of bamboo scrimber in structural applications. Due to the propensity of cracks to propagate, it reduces the load-bearing capacity of the bamboo scrimber material. Therefore, research on the fracture toughness of bamboo scrimber contributes to determining the material’s load-bearing capacity and failure mechanisms, enabling its widespread application in engineering failure analysis. The fracture toughness of bamboo scrimber was studied via the single-edge notched beam (SENB) experiment and compact compression (CC) method. Nine groups of longitudinal and transverse samples were selected for experimental investigation. The fracture toughness of longitudinal bamboo scrimber under tensile and compressive loadings was 3.59 MPa·m^1/2^ and 2.39 MPa·m^1/2^, respectively. In addition, the fracture toughness of transverse bamboo scrimber under tensile and compressive conditions was 0.38 MPa·m^1/2^ and 1.79 MPa·m^1/2^, respectively. The results show that, for this material, there was a significant distinction between longitudinal and transverse. Subsequently, three-point bending tests and simulations were studied. The results show that the failure mode and the force–displacement curve of the numerical simulation were highly consistent compared with the experimental results. It could verify the correctness of the test parameters. Finally, the flexural strength of bamboo scrimber was calculated to be as high as 143.16 MPa. This paper provides data accumulation for the numerical simulation of bamboo scrimber, which can further promote the development of bamboo scrimber parameters in all aspects of the application.

## 1. Introduction

Bamboo scrimber is produced through a series of processes including cutting, splitting, defibering, dipping, drying and hot pressing, using bamboo as the raw material [1,2,3]. This material exhibits numerous advantages compared to regular bamboo [4,5]. Bamboo scrimber boasts superior performance and can be customized to address issues such as susceptibility to failure and other limitations of bamboo [6,7]. Its applications extend beyond construction and encompass furniture, transportation and road safety fencing [8,9,10].

In recent years, there has been some progress in exploring the mechanical properties of bamboo scrimber [11,12,13]. However, the majority of research on bamboo scrimber has focused on its fundamental mechanical properties [14,15,16]. Nevertheless, there is a dearth of research on the fracture toughness of bamboo scrimber. The fracture toughness plays a crucial role in assessing the material’s ability to withstand crack propagation [17,18].

He and Evans et al. [19] conducted tests on birch single-layer boards to assess their type I fracture performance. They improved the fracture toughness of birch composites by reinforcing the adhesive and incorporating glass fiber adhesive. However, only a comparison of the apparent fracture toughness was made between the same samples, and no exact fracture toughness value was measured. Zhou et al. [20] performed four-point bending tests on bamboo scrimber and investigated its damage mechanism. They observed that bamboo scrimber exhibited ductile behavior during bending failure, with brittle fracture occurring in the compressive and tensile zones. Nonetheless, the fracture toughness of bamboo scrimber has not been thoroughly studied. Liu et al. [21] determined the fracture behavior and type I fracture toughness of bamboo scrimber. They verified the effectiveness of a method for directly determining the cohesion parameters by comparing numerical simulations with experiments. Ortega et al. [22] experimentally investigated the tensile and compressive fracture toughness of composite laminates using the compact tension and compression methods. He et al. [23] conducted compact compression experiments on carbon fiber, analyzing the displacement and strain field using the J-integral method to obtain the energy dissipation under different loading rates. The numerical value of the fracture toughness was indirectly obtained through the J-integral method. Li et al. [24] studied the fracture toughness of high-strength steel through three-point bending tests and proposed a relationship between fracture toughness and macro tensile parameters. The fracture toughness and tensile strength exhibited an inverse relationship. 

Based on the previous research on the fracture toughness of materials, this study adopted the single-edge notched beam (SENB) and compact compression (CC) methods to study the fracture toughness of bamboo scrimber in different directions. In addition, through the three-point bending tests combined with a finite element simulation, the simulation parameters were obtained and determined, which are of great significance for the study of the fracture mechanical properties of bamboo scrimber.

## 2. Tensile and Compressive Fracture Toughness Tests

### 2.1. Specimen Design and Manufacture

**Figure 1:**
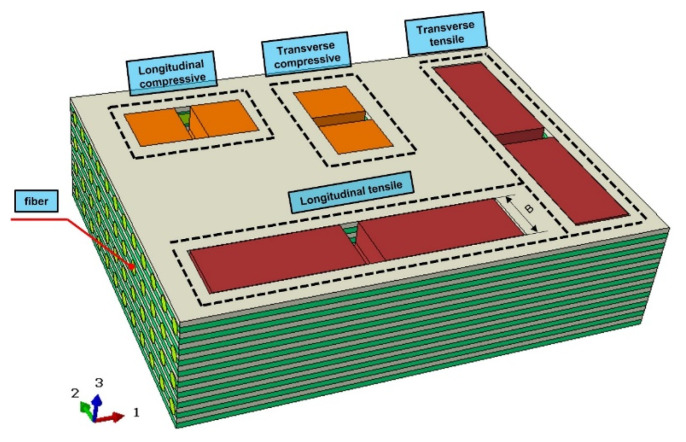
Schematic diagram of bamboo scrimber sheet sampling.

**Figure 2:**
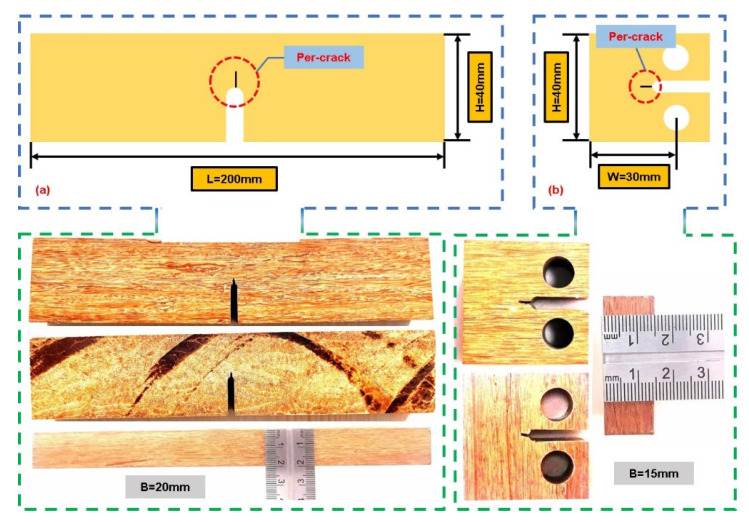
Tensile fracture toughness and compressive fracture toughness: (**a**) longitudinal and transverse single-edge notched beam test specimen dimensions; (**b**) longitudinal and transverse compact compression specimen sizes.

This paper aims to determine the fracture toughness of bamboo scrimber in both the longitudinal and transverse directions under tensile and compressive loading. Bamboo scrimber is produced as a unidirectional sheet using a series of hot-pressing processes [25]. Extensive research has demonstrated significant differences in the mechanical properties between the longitudinal and transverse orientations of unidirectional laminates. Therefore, it is crucial to select and process specimens that differentiate between the transverse and longitudinal stripes on the sheet [26], as depicted in Figure 1. Nine groups of specimens were prepared, each oriented in one of these two directions. The dimensions of the tension and compression specimens are defined by ASTM E399-12 [27] and ASTM E1820-08a [28], respectively, as illustrated in Figure 2. A span length of S = 160 mm was chosen for the experiment, adhering to the standard requirement of 1 ≤ H/B ≤ 4. Consequently, the final dimensions of the tensile fracture toughness specimens were determined as follows: height H = 40 mm, length L = 200 mm, width B = 20 mm and crack length *a* = 20 mm. The crack length was chosen in accordance with the standard range of 0.5 ≤ a/H ≤ 1. To create the initial crack for the tension and compression tests, an 18 mm artificial sharp crack was cut into the specimen. Additionally, a micro-crack measuring 2–3 mm was introduced on the tensile specimen, and a 4–5 mm cut was made on the compression specimen, as depicted in Figure 2. Each specimen was labeled and recorded, with the 9 longitudinal specimens labeled as FT-1~FT-9, and the 9 transverse specimens were named MT-1~MT-9. The length of the pre-crack was also recorded beforehand.

Compression fracture toughness specimens are required to adhere to the standard, which specifies that the specimen width (B) and the distance between the hole center and edge (W) should be chosen within the range of 2 ≤ W/B ≤ 4. In this study, the distance between the hole center and edge was W = 30 mm, and the width (B = 15 mm), length and height (H = 40 mm) were maintained [29]. The 9 longitudinal compression specimens were labeled as FC-1~FC-9, and the 9 transverse compression specimens were denoted as MC-1~MC-9. The respective sizes of each longitudinal and transverse specimen were also recorded in advance.

### 2.2. Fracture Toughness Tests

#### 2.2.1. Test Method

**Figure 3:**
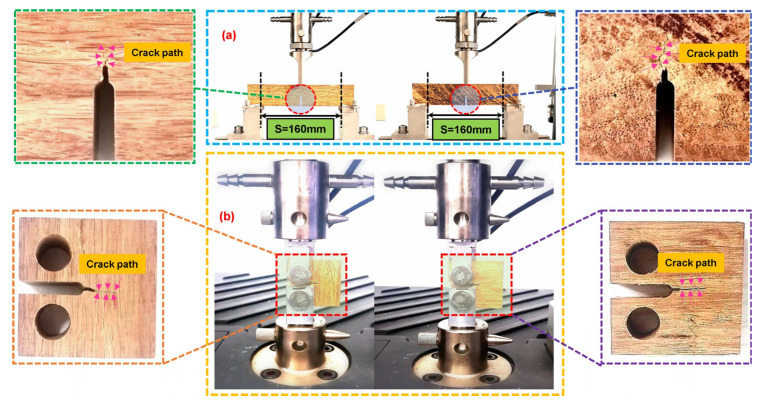
Arrangement of fracture toughness test equipment: (**a**) SENB test on the supports; (**b**) CC in a gripping system.

The fracture toughness of bamboo scrimber was evaluated using the single-edge notched beam (SENB) test and compact compression (CC) test, performed with an electronic universal material testing machine. The test setup and specific details are depicted in Figure 3. Figure 3a illustrates the longitudinal and transverse specimens, and an additional fixture was custom made for the compression fracture toughness tests, as shown in Figure 3b. To ensure a zero initial position of force loading, it is necessary to maintain a gap between the bolt and the specimen during clamping. Therefore, attention should be given to aligning the two nuts properly during tightening, and the upper and lower clamps should also be aligned to avoid nut interference. In the tensile fracture toughness tests, symmetrical positioning of the span and support points with respect to the indenter is crucial, and the indenter should make contact with the specimen before loading to minimize the error between the load and the corresponding loading displacement. Data collection during the experiments was carried out using a force sensor and a built-in displacement sensor. The indenter velocity for the SENB tests was set to 3 mm/min [30], and for the CC tests, it was set to 0.5 mm/min [23]. 

#### 2.2.2. Calculation of Tensile Fracture Toughness

The peak loading (P_Q_) in the SENB tests is obtained from the force–displacement curves, and the fracture toughness (K_IC_) is calculated using Equation (1). Additionally, the energy release rate is calculated using Equation (2). Due to the smaller strain in the thickness direction compared to the length direction, the SENB tests of bamboo scrimber in the longitudinal and transverse directions are classified as plane strain problems. Considering the linearly elastic deformation of bamboo scrimber under longitudinal and transverse tension [1], the critical strain energy release rate is twice the fracture energy, as shown in Equation (3) [31].

The fracture toughness K and energy release rate G of the SENB tests are calculated as follows [32]:

(1) $$K=\frac{P_{Q}}{B\sqrt{W}}\left[2.9-4.6\left(\frac{a}{W}\right)+21.8{\left(\frac{a}{W}\right)}^{2}-37.6{\left(\frac{a}{W}\right)}^{3}+38.7{\left(\frac{a}{W}\right)}^{4}\right]$$

where P_Q_ is the peak force.

(2) $$G=\frac{K^{2}}{E^{\prime }}$$

If $E^{\prime }$ is a plane strain problem, then $E^{\prime }$= E/(1 − μ^2^). If it is a plane stress problem, then $E^{\prime }$ = E. In addition, μ is Poisson’s ratio.

(3) $$G=-\frac{\partial U}{\partial A}=2\gamma$$

In Equation (3), ∂U is the potential energy, ∂A is the increase in crack area, and *γ* is the fracture energy.

#### 2.2.3. The Theory of Compressive Fracture Toughness

**Figure 4:**
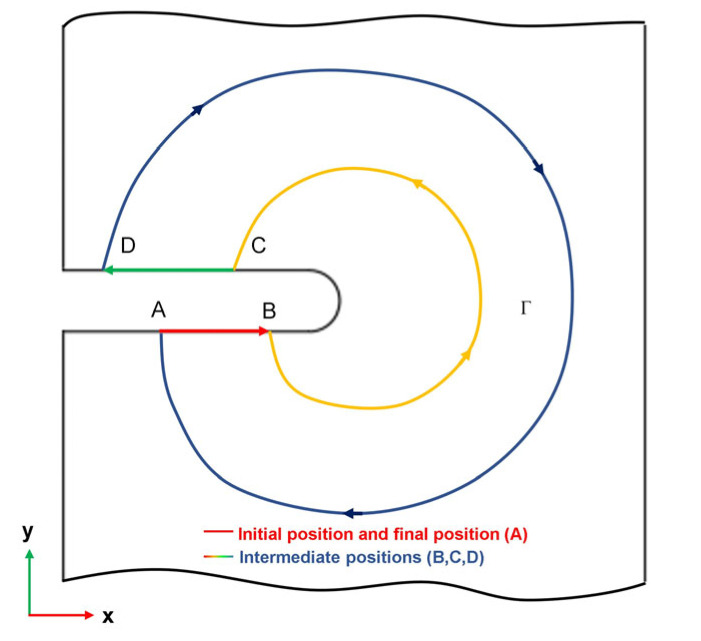
Smooth closed-loop illustration of crack tip.

In the CC tests, the J-integral method is employed to evaluate the energy release rate. The J-integral is defined for a two-dimensional cracked body, as depicted in Figure 4. A random point along the crack is selected, and a continuous closed loop is formed in a counterclockwise direction [33], The J-integral is calculated using Equation (4), as follows:

(4) $$J=\int_{\Gamma }\mathrm{Vdy}-p_{\alpha }\frac{\partial u_{\alpha }}{\partial x}ds$$

where p_α_ is the external loading on the crack surface, V is the strain energy density, u_q_ represents the component of the displacement vector, and ds represents the infinitesimal increment along the integration path Γ [34], which can be defined as

(5) $$V=\int_{0}^{\varepsilon_{ij}}\sigma_{ij}d\varepsilon_{ij}$$

The value of the J-integral is equal to the value of the energy release rate [35]. Assuming that the compressive fracture toughness of bamboo scrimber under compression is treated as linear elasticity, the J-integral has a corresponding relationship with the stress intensity factor K_j_, as given by the following equation:

(6) $$J=\frac{\left(1-\nu^{2}\right)}{E}K_{j}^{2}$$

In addition, the energy release rate is obtained as

(7) $$G_{I}=\frac{K_{j}^{2}}{E}\left(1-\nu^{2}\right)$$

The fracture toughness K can be solved. For the force P_i_ exerted on the compacted samples, K can be calculated as

(8) $$K_{\left(i\right)}=\frac{P_{i}}{{\left(BB_{N}W\right)}^{1/2}}f\left(\frac{a_{i}}{W}\right)$$

(9) $$f\left(\frac{a_{i}}{W}\right)=\frac{\left(2+\frac{a_{i}}{W}\right)\left(0.886+4.64\left(\frac{a_{i}}{W}\right)-13.32{\left(\frac{a_{i}}{W}\right)}^{2}+14.72{\left(\frac{a_{i}}{W}\right)}^{3}-5.6{\left(\frac{a_{i}}{W}\right)}^{4}\right)}{{\left(1-\frac{a_{i}}{W}\right)}^{3/2}}$$

where P_i_ refers to the peak force in the force–displacement curve during the compression process.

The calculation of the J-integral is the sum of elastic-specific work and plastic-specific work, as follows:

(10) $$J=J_{el}+J_{pl}$$

Therefore, the total J-integral can be calculated as

(11) $$J=\frac{K^{2}\left(1-\nu^{2}\right)}{E}+J_{pl}$$

In addition, $J_{pl}$ is calculated as

(12) $$J_{pl}=\frac{\eta A_{pl}}{B_{N}b_{0}}$$

**Figure 5:**
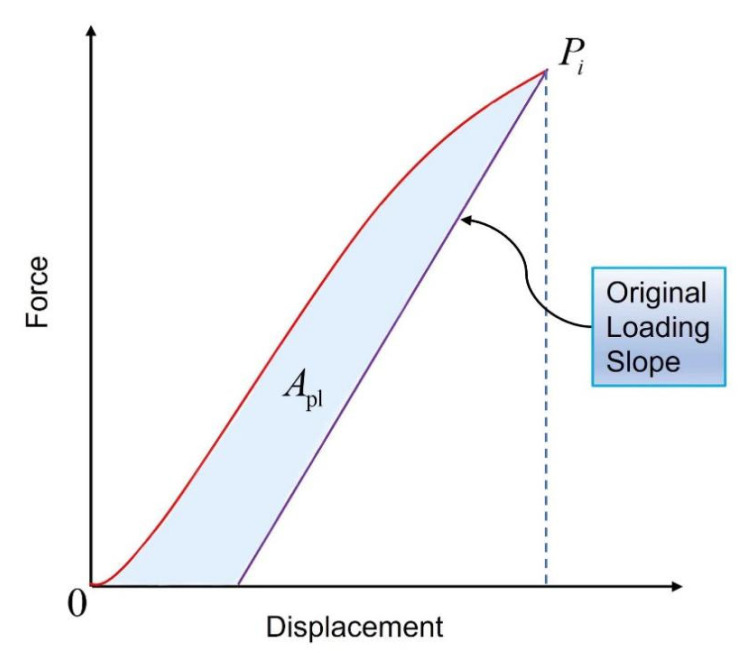
Definition of area for J calculation using the basic method.

In Equation (12), $A_{pl}$ refers to the area enclosed by the initial line segment with the same slope at the points corresponding to the force–displacement curve and maximum loading, as shown in Figure 5. $B_{N}$ is the thickness of the specimen ($B_{N}=B$ in this study); $b_{0}=\left(W-a_{0}\right)$; and $\eta =2+0.522b_{0}/W$.

### 2.3. Experiment Results

#### 2.3.1. Experiment Results of Tensile Fracture Toughness

**Figure 6:**
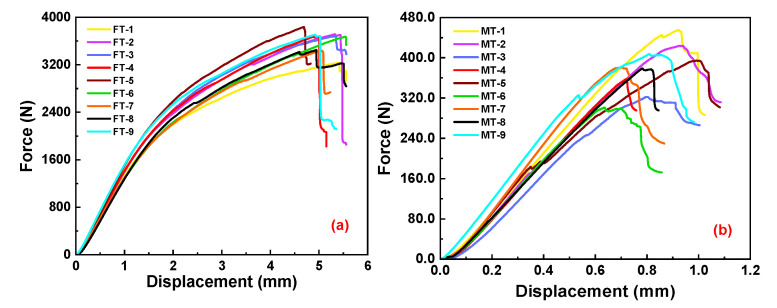
Experimental results of SENB tests: (**a**) longitudinal tensile force–displacement curves; (**b**) transverse tensile force–displacement curves.

The results of the tensile fracture toughness tests for both the longitudinal and transverse specimens are presented in Figure 6a and Figure 6b, respectively. Bamboo scrimber exhibits elastic behavior during the initial stage. However, as the applied loading approaches the peak force, it deviates from its linear response. As the applied force approaches the peak force, the curve no longer exhibits a linear behavior. At this stage, cracks may occur within the specimen, absorbing energy and impeding crack propagation. Upon reaching the ultimate bearing capacity, the samples fracture, leading to a steep decline in the curves. The peak force of the longitudinal samples from each group are extracted and incorporated into Equation (1), along with the measured value of the width (B), height (W) and crack length (a) before the tests. The average longitudinal tensile fracture toughness is 3.59 MPa·m^1/2^, whereas the average transverse tensile fracture toughness is 0.38 MPa·m^1/2^. The longitudinal tensile fracture toughness of bamboo scrimber is approximately 9.45 times higher than the transverse tensile fracture toughness, suggesting superior load-bearing capacity in tension for bamboo scrimber. In this study, the Young’s modulus E_1_ = 11,212 MPa, E_2_ = 2561 MPa and Poisson’s ratio μ = 0.304 are adopted from [36]. The obtained longitudinal and transverse fracture toughness values for bamboo scrimber are inserted into Equation (2). The longitudinal and transverse tensile energy release rates are 1043.26 J/m^2^ and 51.17 J/m^2^, respectively. Consequently, the longitudinal and transverse tensile fracture energy values are 521.63 J/m^2^ and 25.59 J/m^2^, respectively, because the energy release rate is twice the fracture energy. The longitudinal tensile energy release rate for bamboo scrimber is 992.09 J/m^2^ higher than the transverse tensile energy release rate.

#### 2.3.2. Test Results of Compressive Fracture Toughness

**Figure 7:**
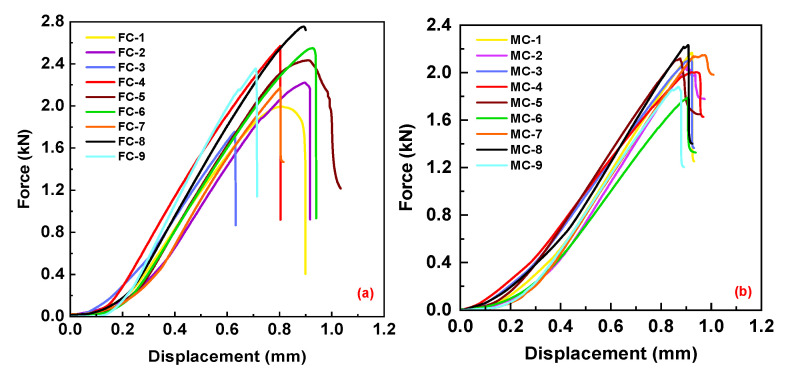
Experimental results of CC tests: (**a**) longitudinal compression force–displacement curves; (**b**) transverse compression force–displacement curves.

The experimental results of the longitudinal and transverse compressive fracture toughness of bamboo scrimber are shown in Figure 7a and Figure 7b, respectively. The force–displacement curve exhibits a small non-linear segment at the beginning, which is attributed to the initial minor slippage occurring during the clamping of the specimen in the experiment. Subsequently, the displacement shows an almost linear relationship with the increasing force. Moreover, the test results for each group of specimens exhibit a high level of consistency. The peak loading from the longitudinal and transverse tests, along with the measured values of the width (B) and the distance between the hole center and edge (W) before the tests are substituted into Equations (8) and (9). The average longitudinal fracture toughness is 2.39 MPa·m^1/2^, and the average transverse fracture toughness is 1.79 MPa·m^1/2^. The longitudinal compressive fracture toughness of bamboo scrimber is approximately 1.34 times greater than the transverse compressive fracture toughness. These results indicate a lower fracture toughness in the transverse direction compared to the longitudinal direction, highlighting a weaker load-bearing capacity under compressive forces in the transverse direction. In this study, the J_el_ value can be obtained by substituting the calculated fracture toughness into Equation (11). Then, the value of A_pl_, b_0_, and η of each group is calculated and substituted into Equation (12) to calculate the J_pl_. Then, the corresponding J_el_ + J_pl_ is calculated, and the energy release rate can also be obtained. The average longitudinal energy release rate is 324.67 J/m^2^, and the average transverse energy release rate is 222.45 J/m^2^. The longitudinal compressive energy release rate for bamboo scrimber is 102.22 J/m^2^ higher than the transverse compressive energy release rate.

## 3. Simulation and Experimental Verification

### 3.1. Three-Point Bending Tests

**Figure 8:**
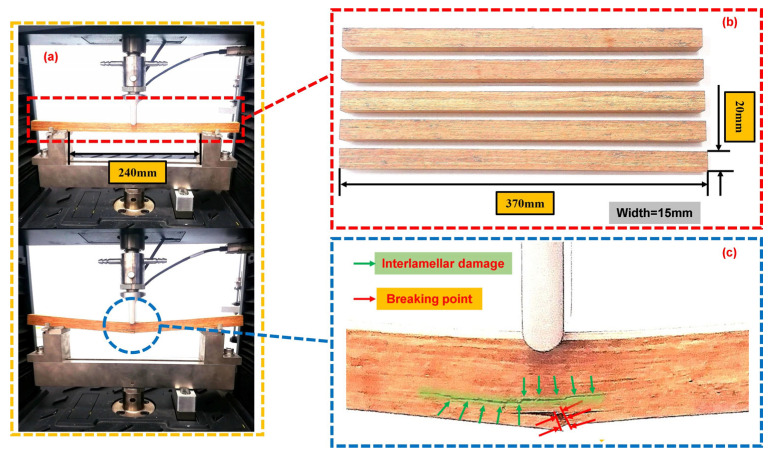
Three-point bending tests: (**a**) layout of three-point bending specimens; (**b**) three-point bending specimen parameters; (**c**) failure pattern of three-point bending specimens.

In order to validate the energy release rate damage parameter used in ABAQUS for bamboo scrimber, the results of the three-point bend tests were compared with the corresponding numerical simulations. The fracture of bamboo scrimber in three-point bending represents a complex damage mechanism, involving the release of stored energy within the material during the fracture process [37]. Five longitudinal bamboo scrimber samples, each with a width of 15 mm, were prepared and labeled as QW-1 to QW-5. Their dimensions are illustrated in Figure 8b. The distance between the two supports was 240 mm, and the loading speed was set at 10 mm/min following the guidelines of GB/T 1936.1-2009 [38]. The specimen placement is illustrated in Figure 8a. The failure pattern of the three-point bending specimens at the end of loading is shown in Figure 8c. The primary failure mode observed in bamboo scrimber is brittle fracture, with cracks propagating inward from the fracture surface. Due to defects in the lamination process, cracking occurs gradually between the layers of bamboo scrimber, eventually resulting in a loss of load-bearing capacity.

**Figure 9:**
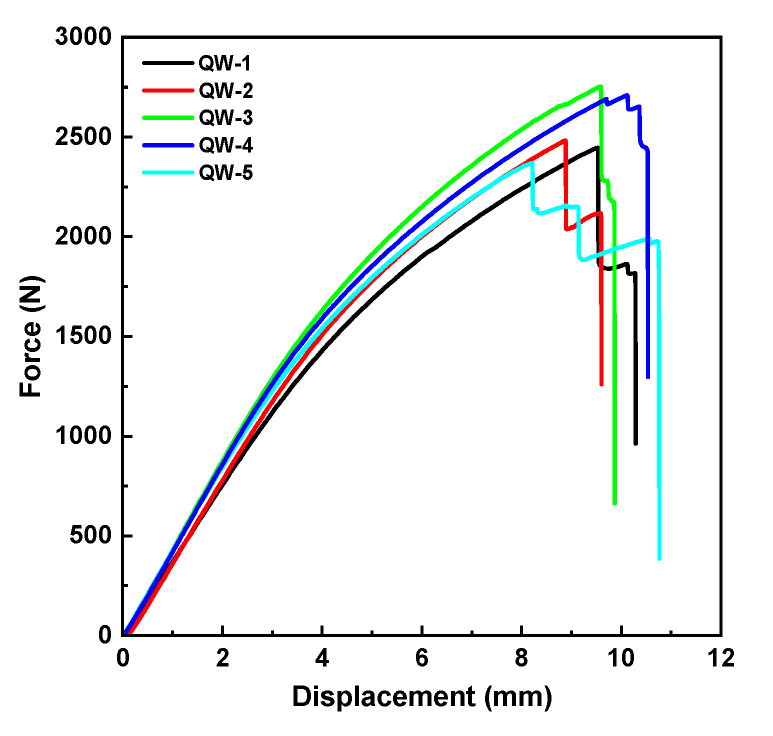
Force–displacement curves of three-point bending tests.

Figure 9 displays the force–displacement curves. The graph clearly indicates that, in the initial stage, the curve exhibited linear growth, with the bamboo scrimber specimen experiencing slight bending during the loading process. At the point where the bamboo scrimber reached its ultimate strength, corresponding to the peak force, the specimens underwent brittle fracture. Subsequently, the curve began to decline, indicating a significant loss of load-bearing capacity. The fracture loading of each curve is recorded in Table 1. In addition, the average failure force was calculated as 2552.10 N. The width, height, fracture force and span, measured prior to the tests, were used as inputs in Equation (13) to calculate the bending strength [39]. The average value obtained was 142.78 MPa.

(13) $$\sigma_{b}=\frac{3P_{\max}l}{{2bh}^{2}}$$

where $P_{\max}$ is the failure loading, and the unit is N; l is the span between the two supports, and the unit is mm; and b and h are the width and height of the specimens, respectively, and the unit is mm.

### 3.2. Numerical Results

**Figure 10:**
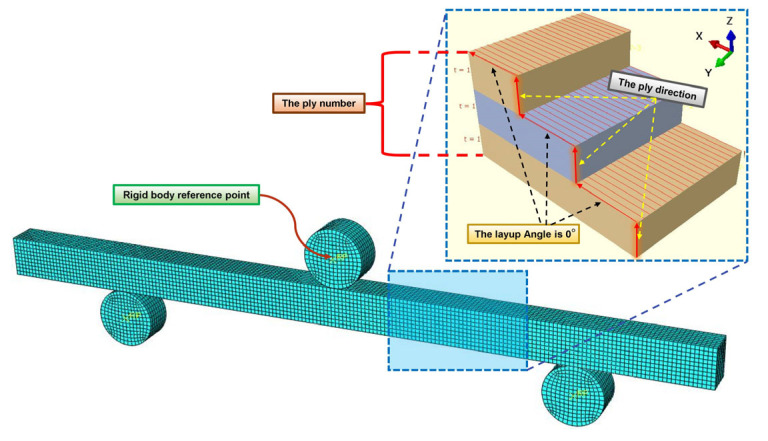
Model and mesh of bamboo scrimber three-point bending tests.

The ABAQUS nonlinear explicit analysis was employed in this study to simulate the three-point bending tests. By incorporating the experimentally determined energy release rate parameters into ABAQUS, the congruity between the deformation patterns and force–displacement curves obtained from the numerical simulations and experimental results can substantiate the reliability of the numerical model and the applicability of the experimental result parameters. Bamboo scrimber consists of the fiber reinforced phase and the matrix phase, which is defined as bamboo fiber reinforced composite, and the failure process was simulated by using the Hashin failure criterion [40]. The hexahedral sweeping meshing technique was employed for grid partitioning. Due to the fact that actual bamboo scrimber is manufactured by hot-pressing laminates, the layers were arranged along the thickness direction based on the geometric shape of the bamboo scrimber [41]. The simulation model and mesh division of the three-point bending tests are shown in Figure 10. The model size was 370 mm × 15 mm × 20 mm. Table 2 summarizes the in-plane and out-of-plane shear parameters of bamboo scrimber. The layup angle was set at 0°, and the layering direction followed the Z-axis. The basic Hashin damage parameters are provided in Table 3. The element type was SC8R, and the total number of 14,880 elements were produced. The element deletion was set. Element deletion can be employed to simulate damage. As the loading progresses, the elements reach the critical strain energy release rate, leading to failure and automatic deletion of the elements. The support base and indenter both had a cylindrical shape, with a diameter of 20 mm and a height of 15 mm. The two support bases and the upper indenter were assigned as rigid bodies, with the element type set as C3D8R. Fully fixed boundary conditions were applied to the two supports, and a downward displacement of 10 mm was imposed on the indenter. 

### 3.3. Comparison of Results

**Figure 11:**
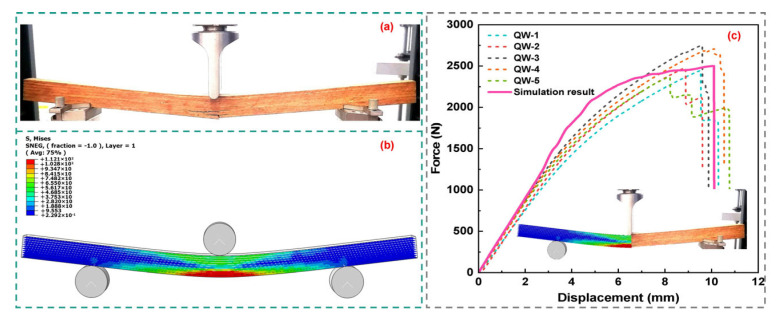
Comparison of test and simulation: (**a**) three-point bending test failure diagram; (**b**) three-point bending simulation damage nephogram; (**c**) comparison of three-point bending test force–displacement curves.

The results of the three-point bending tests and numerical simulations are presented in Figure 11a and Figure 11b, respectively. Figure 11c illustrates an enlarged comparison of the failure patterns observed in both the test and simulation, revealing their near-identical nature. The numerical simulation and experimental force–displacement curves are presented in Figure 11. It can be seen that the simulation results are similar to the test results. Furthermore, the simulation yielded a peak force of approximately 2500.64 N, exhibiting an error of approximately 2.02% when compared to the peak force observed in the experimental results. The ultimate bending strength was calculated to be 150.04 MPa by substituting the model dimensions and the peak force obtained from the numerical simulation into Equation (13). As a result, the outstanding agreement between the simulation results and experimental findings verified the reliability of the numerical model and the applicability of the experimental parameters.

## 4. Conclusions

This study aimed to experimentally investigate the fracture toughness and energy release rate of bamboo scrimber, as well as to determine the simulation parameters for the material. The following conclusions can be drawn from this study:

The tensile fracture toughness of bamboo scrimber was determined using the SENB test method for both the longitudinal and transverse directions. The average longitudinal tensile fracture toughness of bamboo scrimber was 3.59 MPa·m^1/2^, and the average transverse tensile fracture toughness was 0.38 MPa·m^1/2^. Similarly, the average longitudinal tensile energy release rate was 1043 J/m^2^, whereas the average transverse tensile energy release rate was 51.17 J/m^2^. The compressive fracture toughness of bamboo scrimber in both the longitudinal and transverse directions was determined using the CC method. The average longitudinal compression fracture toughness of bamboo scrimber was 2.39 MPa·m^1/2^, and the average transverse compression fracture toughness was 1.79 MPa·m^1/2^. Similarly, the average longitudinal compression energy release rate was 324.67 J/m^2^, whereas the average transverse compression energy release rate was 222.45 J/m^2^. The longitudinal tensile fracture toughness of bamboo scrimber was significantly higher than its compressive fracture toughness, demonstrating superior tensile resistance. The higher values of longitudinal tensile and compressive energy release rates compared to the transverse direction indicate that bamboo scrimber possesses greater strength, stiffness and deformability along the longitudinal axis, while exhibiting relatively weaker behavior in the transverse direction. Overall, this finding holds significant implications for the application and engineering design of bamboo scrimber, allowing for the better utilization of its performance characteristics. Specifically, in situations where resistance to tensile stresses is crucial, bamboo scrimber can leverage its strengths, facilitating the selection of suitable application orientations and design parameters.

Three-point bending tests were conducted to obtain the force–displacement curves of bamboo scrimber. The average bending strength of bamboo scrimber was calculated as 142.78 MPa using the fracture load and specimen parameters. To determine the constitutive parameters of failure Hashin damage in ABAQUS, the measured parameters were introduced into the three-point bending simulation. The force–displacement curves obtained from the numerical simulations closely matched the experimental force–displacement curves. It should be pointed out that the failure load of the simulated force–displacement curves can be used to calculate the flexural strength. The error of the calculated results was about 5.08%, which indicates that the numerical simulation could simulate the test well. This demonstrates the reliability of the numerical model and the applicability of the experimental result parameters. Furthermore, the experimental result parameters can be utilized for subsequent numerical analyses and predictions. However, the numerical model has its limitations, such as the possibility of manufacturing defects in the resin impregnation during the bamboo scrimber process. The numerical simulations were conducted under ideal conditions and could not replicate the actual situation completely. Therefore, there is still room for improvement in the generation of random defects in the numerical simulations.

## Figures and Tables

**Table 1:** Three-point bending test parameters and results.

Number	Failure Loading (N)	Bending Strength (MPa)
QW-1	2445.84	136.10
QW-2	2481.94	139.57
QW-3	2753.35	151.44
QW-4	2370.31	134.33
QW-5	2709.09	152.47
Average	2552.11	142.78

**Table 2:** Shear parameters in and out of the bamboo scrimber plane [36].

G_12_ (MPa)	G_13_ (MPa)	G_23_ (MPa)
1418	1418	749

**Table 3:** Basic Hashin parameters.

Longitudinal			Transverse		
Ultimate Tensile Strength	Compressive Strength	Ultimate Shear Strength	Ultimate TENSILE Strength	Compressive Strength	Ultimate Shear Strength
113.86 MPa	63.14 MPa	4.80 MPa	21.51 MPa	8.19 MPa	24.25 MPa

## Data Availability

Not applicable.
